# Health Literacy in Latin America and the Caribbean: A Comprehensive Scoping Review of Research Developments

**DOI:** 10.1007/s10935-026-00922-w

**Published:** 2026-05-25

**Authors:** Kristine Sørensen, Lilliana Villa-Vélez, Stefanie Harsch, Virginia Visconde Brasil

**Affiliations:** 1 Global Health Literacy Academy & Aalborg University, Risskov, Denmark; 2 Faculty of Medicine, University of Antioquia, Medellín, Colombia; 3 University of Education Freiburg, Freiburg, Germany; 4 Federal University of Goias, Goiania, Brazil

**Keywords:** Health literacy, Latin America, South America, The Caribbean, Scoping review

## Abstract

Health literacy is critical for reducing inequality and promoting effective implementation of health services and better health outcomes. The health literacy agenda is gaining momentum worldwide, including in Latin America and the Caribbean. However, a comprehensive overview of research developments in the region is lacking. This study aimed to explore the state of the art of health literacy research in Latin America and the Caribbean. A scoping review was conducted in January 2024 drawing upon data from the databases: La Referencia, BVS, PubMed, Web of Science, CINAHL, PsycINFO, and ERIC. The search results were reviewed in Rayyan by all co-authors. The title and abstracts were screened, based on inclusion and exclusion criteria followed by extraction, analysis and synthesis of data according to the research objectives. The search yielded 375 publications including 289 peer-reviewed articles and 86 dissertations and theses. The sample included quantitative (73.9%), qualitative (13.9%), mixed-methods (5.9%), and bibliographic (1.9%) studies and other types of scientific publications (4.5%). Two thirds of the countries published health literacy research. Most originated from Brazil, followed by Mexico, Chile, Peru, and Colombia. There was a marked increase in publications from 2017 onward, reaching a peak in 2022. The most frequently cited definitions were by Sørensen et al. (Sørensen et al in BMC Pub Health 12:80, 2012 and the WHO Health Promotion Glossary (World Health Organization (WHO). (1998). Health promotion glossary.). The prominent research topics included oral health literacy and non-communicable diseases. Other topics include e.g., general health literacy, communicable diseases, functional health literacy, and healthcare literacy. Health literacy was measured 312 times with a variety of established and newly developed instruments. This scoping review highlights progress and challenges concerning health literacy research in Latin America and the Caribbean. The active health literacy research community adopts multiple research strategies, themes and multidisciplinary approaches for adapting health literacy interventions to diverse populations. Brazil and its vibrant health literacy community can inspire the development of health literacy in the region. Ongoing research, policy innovation, and community-based action are needed to maximize health literacy’s potential for improving health services, public health, and wellbeing in the region.

## Background

Health literacy has gained global attention as a critical determinant of health outcomes, equity, and system performance (The Economist Intelligence Unit Limited, 2021). A large body of research covered health literacy developments in different world regions (Baccolini et al., 2021; Elyamani et al., 2021; Rajah et al., 2019; Sørensen et al., 2024; Wikkeling-Scott et al., 2019) or among various population groups (Bröder et al., 2017; Sarhan et al., 2023; Sarr et al., 2025; Ward et al., 2019). However, the scope, scale, and characteristics of health literacy in Latin America and the Caribbean remain underexplored and fragmented (Arrighi et al., 2022). The Latin American and Caribbean geographical region is characterized by significant social, cultural, and economic heterogeneity, entrenched health inequalities, and systemic barriers to healthcare access (OECD & The World Bank, 2023). The aforementioned challenges are further compounded by the presence of multilingual populations, educational disparities, and varied health system capacities. Consequently, the role of health literacy becomes increasingly relevant in reducing inequalities and promoting improvements in health services (Baumeister et al., 2021). The adoption of health literacy is a human-rights concern which calls for action in policy, research, and practice ( M-POHL., 2023; Sørensen, 2024).

Health literacy entails the “knowledge, motivation and competencies to access, understand, appraise and apply information to form judgement and make decisions in everyday life concerning healthcare, disease prevention and health promotion to maintain and promote quality of life during the life course” (Sørensen et al., 2012). It is a critical empowerment strategy for people to manage their health and well-being, seek out information, take responsibility, and make sound decisions in the context of everyday life – at home, in the community, at the workplace, in the healthcare system, the marketplace, and the political arena (Kickbusch, 2005). As such, the environment plays a vital role in how health literacy unfolds based on the support from institutions and organizations. This perspective is called organizational health literacy, defined as “the degree to which an organization implements policies, practices, and systems that make it easier for people to navigate, understand, and use information and services to take care of their health” (Agency for Healthcare Research and Quality et al., 2012).

However, health literacy limitations threaten to undermine the effectiveness and equity of health systems and are considered a key source of economic inefficiency (Rasu et al., 2015; The Economist Intelligence Unit Limited, 2021). Extrapolated national estimates from the U.S. show that the annual costs for prescription medication for adults with limited health literacy alone could reach about $172 billion (Rasu et al., 2015). The detrimental impact of limited health literacy hampers individuals’ opportunities to take control of their health and well-being, organizations’ ability to provide efficient care, and the societal implications for equity and sustainability (de Jesus et al., 2024). From a societal point of view, improving limited levels of health literacy by just 25% could save $303 billion a year across 40 countries, according to Economic Impact (Economist Impact, 2025).

### Latin America and the Caribbean from a Regional Perspective

Latin America and the Caribbean region hosts a population of approximately 663 million people living in 33 countries (Economic Commission for Latin America and the Caribbean (ECLAC), 2024; OECD & The World Bank, 2023), extending from the Yucatán Peninsula in the north, traversing the Isthmus of Panama, and extending throughout the South American continent to Tierra del Fuego in the south. In addition to this extensive mainland territory, the region includes numerous island nations, particularly within the Caribbean, encompassing the Greater and Lesser Antilles. The most populated countries include Brazil, Mexico, Colombia, Argentina, Peru, and Venezuela.

The geographic classification of the Americas is conventionally divided into three subregions: North America, Central America, and South America. Conversely, Latin America and the Caribbean signify a cultural and socio-political regionalization, predominantly by shared historical, linguistic, and economic characteristics rather than physical features (Kent, 2016). The region has historically been inhabited by diverse Indigenous civilizations and underwent significant transformation following European colonization, predominantly by Spanish and Portuguese explorers. As a result, Latin America and the Caribbean is linguistically and culturally diverse, with major languages including Spanish and Portuguese; other languages are French, Dutch, English, and various Indigenous languages such as Quechua, Guaraní, Aymara, Náhuatl, among others (The World Bank, 2015; UNICEF & FUNPROEIB Andes, 2009). Today, the region’s demographic profile reflects a rich blend of Indigenous, African, and European ancestries, contributing to its complex cultural identity. Notwithstanding the notable economic advancements witnessed in countries such as Brazil, Chile, Mexico, and Uruguay, Latin America and the Caribbean remain constituted mainly by low and middle-income countries and are characterized by profound social and economic disparities (Pan American Health Organization 2024; 2025).

### Exploring Health Literacy in Latin America and the Caribbean

To date, only a few scientific publications have explored health literacy in Latin America and the Caribbean from a general point of view. The first was a narrative review, published in 2021, which sought to examine and explore the state of health literacy and related efforts in Latin America. However, this review did not employ a systematic approach to detect any study on health literacy in Latin America and is therefore limited both in its methodological, comprehensive, and temporal scope (Arrighi et al., 2022). Another scoping review protocol (Lima et al., 2023) focused on assessment strategies to measure health literacy in Latin America and the Caribbean up to July 2023, but not the wider scope of health literacy research developments. Despite the growing international literature on health literacy, there is limited synthesis and comparative analysis of how the concept has been developed, adapted, and applied in the Latin American and Caribbean contexts. As such, there is a clear need to map existing research to identify gaps, highlight regional innovations, and inform future directions.

For this purpose, an appropriate methodological approach was to conduct a scoping review, given its utility in systematically examining emerging fields, capturing the breadth of literature, and clarifying conceptual boundaries (Munn et al., 2018). This approach allows the identification of research trends and major themes regarding the development and implementation of health literacy initiatives in Latin America and the Caribbean. The insights may also support efforts to strengthen regional collaborations, tailor interventions to local realities, and ensure that health literacy is a tool for equity and empowerment across diverse Latin American services, settings, and systems.

This study deliberately pursues a whole-of-Latin-America-and-the-Caribbean approach to gain a wide perspective of the scope and scale of health literacy developments in Latin America and the Caribbean. For this reason, the research question focused on "How has health literacy research evolved in Latin America and the Caribbean, and what are the prevailing historical developments, geographical distribution, themes, methodologies, gaps, and opportunities?" The findings of this scoping review may inform and inspire further expansion of the health literacy agenda across the region.

## Methods

### Study Design and Research Group

The study design was informed by Arksey and O’Malley, the PRISMA Guidelines for literature reviews, as well as the JBI recommendations, allowing for systematic and transparent data identification, selection, synthesis, and appraisal of research-related literature (Arksey & O’Malley, 2005; Moher et al., 2010; Peters et al., 2024; Tricco et al., 2018). The review was conducted by a multi-national and multi-disciplinary research group within and beyond Latin America, from Brazil, Colombia, Denmark and Germany proficient in English, Portuguese, Spanish, and French, and trained in public health, nursing, medicine, global health, adult education, and applied linguistics. All are experts in health literacy.

### Data Collection

The data was derived from La Referencia, Biblioteca Virtual en Salud (BVS), PubMed, Web of Science, CINAHL, PsycINFO, and ERIC. The search string was composed of the search terms related to the three components of the population-concept-context (PCC) model. Firstly, the concept was “health literacy” OR “alfabetización para la salud” OR “alfabetización en salud” OR “letramento em saúde” OR “literacia em saúde” OR "letramento funcional em saúde" OR “alfabetização em saúde” OR “alfabetização funcional em saúde”. Secondly and thirdly, the ‘context’ and ‘population’ comprised “Latin America” or “South America” or the names of the 35 countries listed by Pan American Health Organization (PAHO), excluding the USA and Canada. (OECD & The World Bank, 2023; PAHO, 2024). The search encompassed articles, theses, and dissertations written in English, Portuguese, Spanish, and French that explicitly referenced the concept of health literacy in their titles or abstracts. The search was not constrained by a specific temporal limitation. The exact search strategy, the full search string used on different databases, and a description of the databases can be found in Supplement 1.

### Data Selection

The data collection was completed on 26 January 2024. All records retrieved through the searches were imported into Rayyan, a specialized literature review software (Ouzzani et al., 2016), and duplicates were removed. The four co-authors independently reviewed titles and abstracts to determine their eligibility. The full texts of potentially relevant studies were assessed against the inclusion and exclusion criteria. Disagreements were addressed through group discussion and consensus-building (Table 1).

**Table 1:** Shows the inclusion and exclusion criteria applied.

	Inclusion	Exclusion
Population	All people living in Latin America and the Caribbean (natives & migrants) OR English, Spanish, Portuguese, and French	Peoples from Latin America or the Caribbean living outside the region or a heterogeneous group of people from various countries without distinguishing the countries
Concept (“health literacy”)	Health literacy (translated in four languages) and the concept is part of the research question, aim, objectives, or findings sections	Only health knowledge OR HL was only referred to in the introduction, conclusion, or recommendation
Context	Country or city from Latin America or the Caribbean OR multiple countries, including one country from Latin American or the Caribbean , and the countries are clearly distinct OR Program/intervention from and based in Latin America or the Caribbean (e.g., Family Health Strategy)	Outside Latin America or the Caribbean or a study including multiple countries, but the data of the countries cannot be clearly distinguished
Type of publication	Research articles, master’s or doctoral theses, government policies, or commentaries registered in scientific databases	Review articles

### Data Sorting

The data was extracted and sorted inductively and applied to a standardized data extraction form developed in a Google Spreadsheet by the authors to reflect the research question. The following key variables were included: author(s), year of publication, country, study design, population, aim, health literacy definition or framework, measurement tools, key themes, findings, and recommendations. The process of data charting was executed by all four co-authors and was meticulously reviewed for accuracy.

### Synthesis and Reporting of Data

The data were synthesized and presented in tabular, graphical, and narrative formats. The findings were organized according to geographic distribution, historical trends, definitions, themes, measurements, and methodological approaches. A quality assessment of sources was not conducted, as it is not a requirement of scoping reviews. The authors followed the PRISMA-ScR checklist for reporting the review, see Supplement 2 (Tricco et al., 2018).

### Study Registration

The scoping review was registered at the Open Science Framework (Brasil et al., 2025) https://doi.org/10.17605/OSF.IO/NWSZY.

## Results

**Fig. 1:**
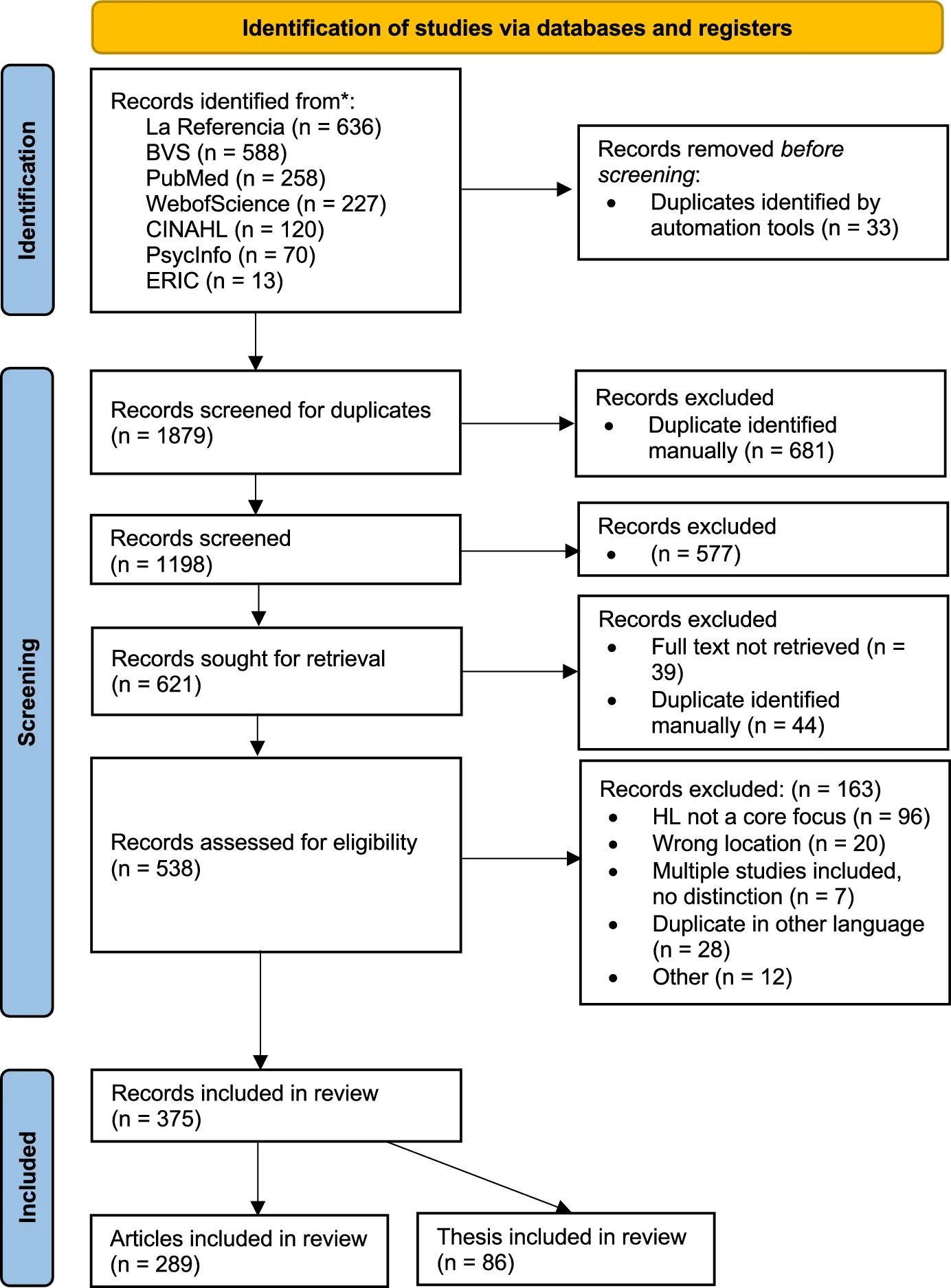
The selection process of publications included in the scoping review of health literacy research development in Latin America and the Caribbean (Page et al., 2021)

The systematic search of scientific publications yielded a total of 1,912 records. Following the removal of duplicates (33 identified by automated tools and 681 records identified manually), the titles and abstracts of 1,198 records were screened for eligibility. Of these, 621 records were sought for retrieval. However, 39 records were inaccessible to the research team, and an additional 44 records were excluded as they were identified as duplicates of records already included in one other language (either English, Spanish or Portuguese), a double check of duplicates was performed, using Rayyan and manually. The full texts of 538 records were assessed for eligibility, resulting in the selection of 375 publications that met all inclusion criteria. Publications were excluded if they did not focus on health literacy, did not cover Latin America and the Caribbean (e.g., Latinos living in the USA), or contained multiple countries that lacked a clear distinction of data related to Latin America or the Caribbean. See Supplement 3 for the entire list of included publications. The selection process is illustrated in the PRISMA-ScR flow diagram (Fig. 1).

The final sample consisted of 375 publications, of which 289 were peer-reviewed articles and 86 were dissertations and theses. The sample included 278 quantitative studies and 52 qualitative studies. Twenty-two studies used a mixed-methods approach. Additionally, there were seven bibliographic studies and 17 miscellaneous publications, such as commentaries, reflections, and descriptions of interventions (see Supplement 4).

### Review of Geographical Origin

**Fig. 2:**
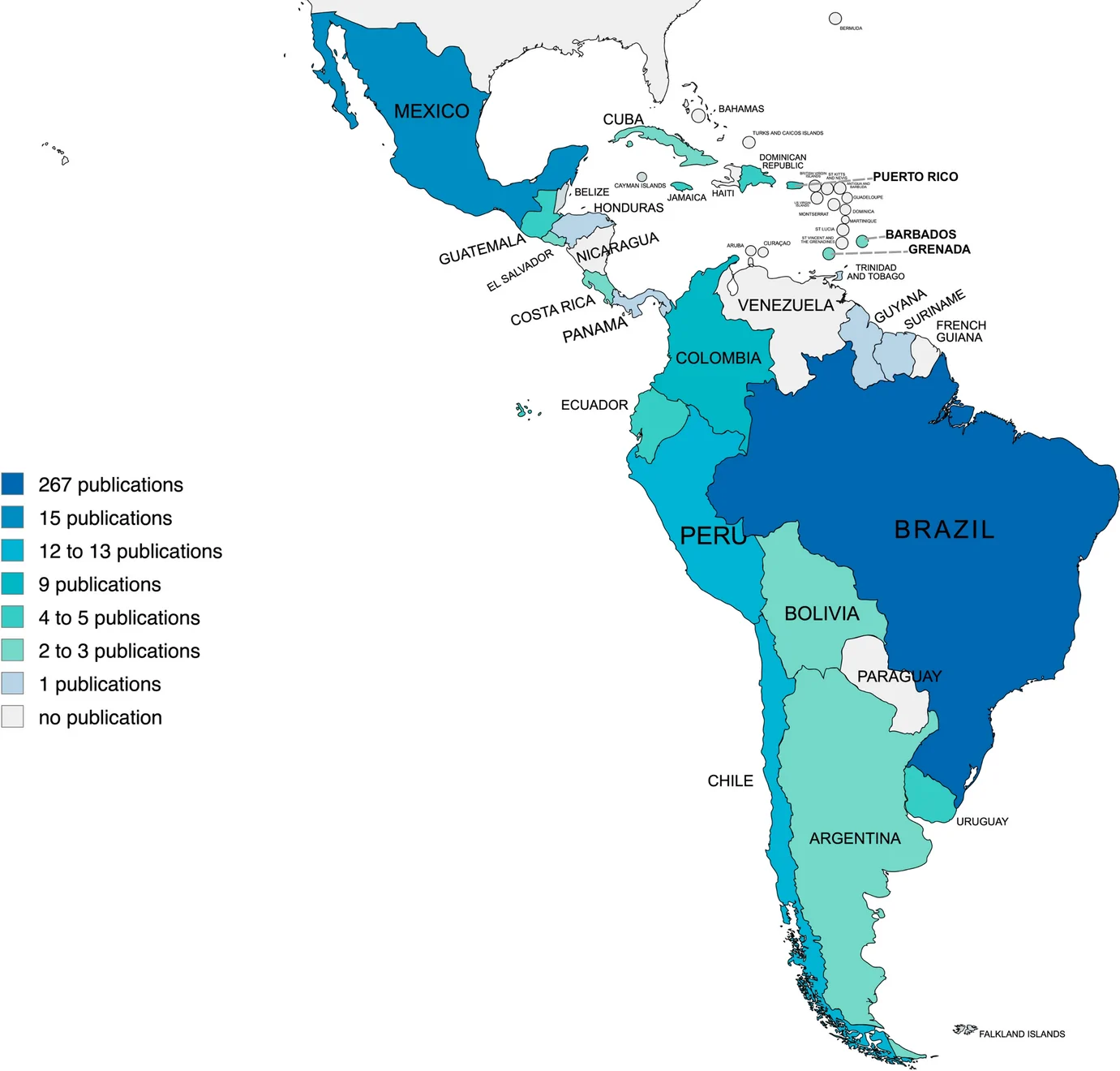
Map of the geographical distribution of health literacy research in Latin America 2009–2024 generated with support by MapChart

Figure 2 illustrates the results regarding geographical distribution. The review revealed that most publications originated from Brazil (71.2%), followed by Mexico (4.2%), Chile (3.5%), Peru (3.2%), and Colombia (2.4%). In addition, 12 multi-country publications were identified, reflecting collaborative efforts across the region. Notably, no publications were identified in 11 of the 33 countries, which highlights the existing gaps in the regional evidence base on health literacy.

### Review of Historical Trends

**Fig. 3:**
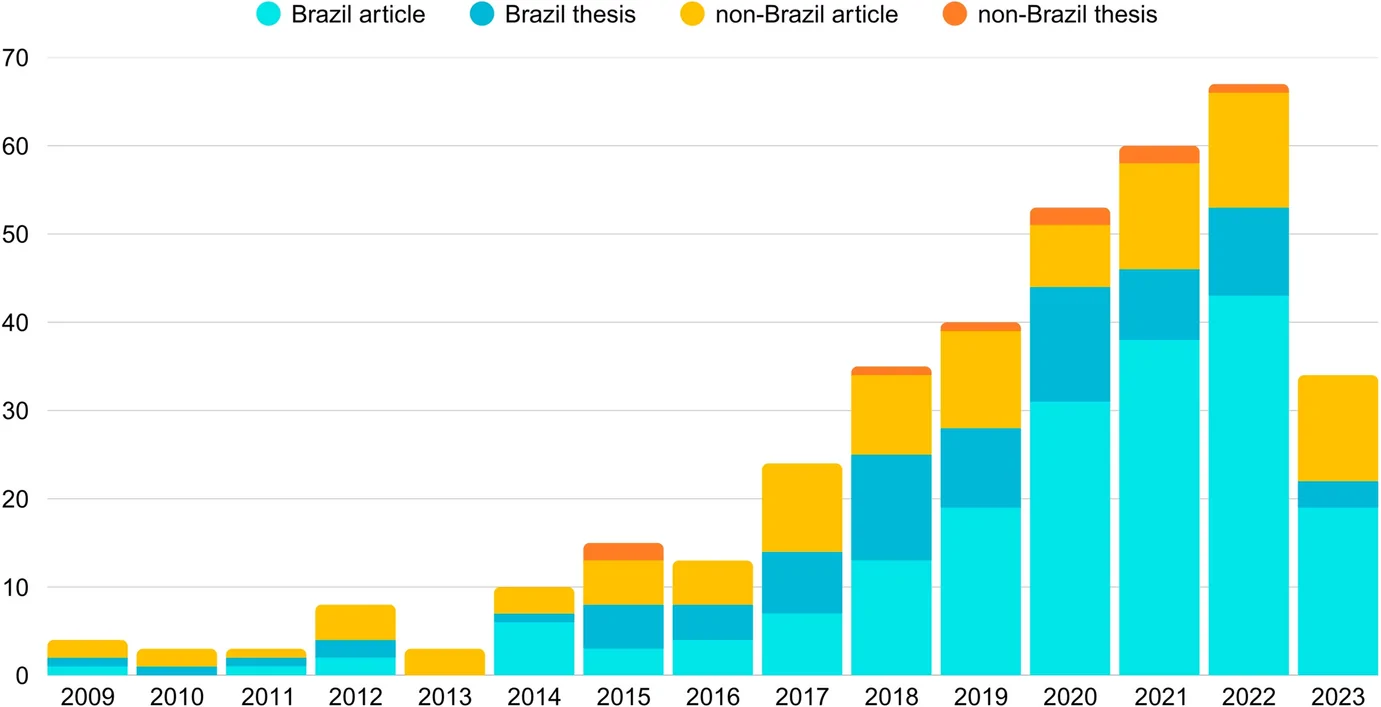
Historical trends of health literacy research publications in Latin America and the Caribbean 2009–2023

The earliest occurrence of scientific publications on health literacy in Latin America and the Caribbean was in 2009. A marked increase in publications can be observed from 2017 onward, reaching a peak in 2022, particularly in Brazil. A variety of trends can be identified through an examination of the data in three distinct ways: firstly, by considering the aggregate data set; secondly, by isolating Brazil from the other countries; and thirdly, by differentiating between scientific articles and theses/dissertations, as illustrated in Fig. 3.

### Review of Health Literacy Definitions

The review revealed that 75.2% of the publications presented a definition or description of health literacy using primary or secondary sources, whereas 24.8% did not specify any descriptive sources. The most frequently used definitions were by Sørensen et al. (2012), cited 60 times, and the WHO Health Promotion Glossary (1998), cited 52 times. Other recurrent health literacy descriptions referred to Nutbeam (2000, 2008), the Institute of Medicine (Nielsen-Bohlman et al., 2004), Ratzan (2001), and Jorm’s work on mental health literacy (1997).

### Review of Health Literacy Topics

**Fig. 4:**
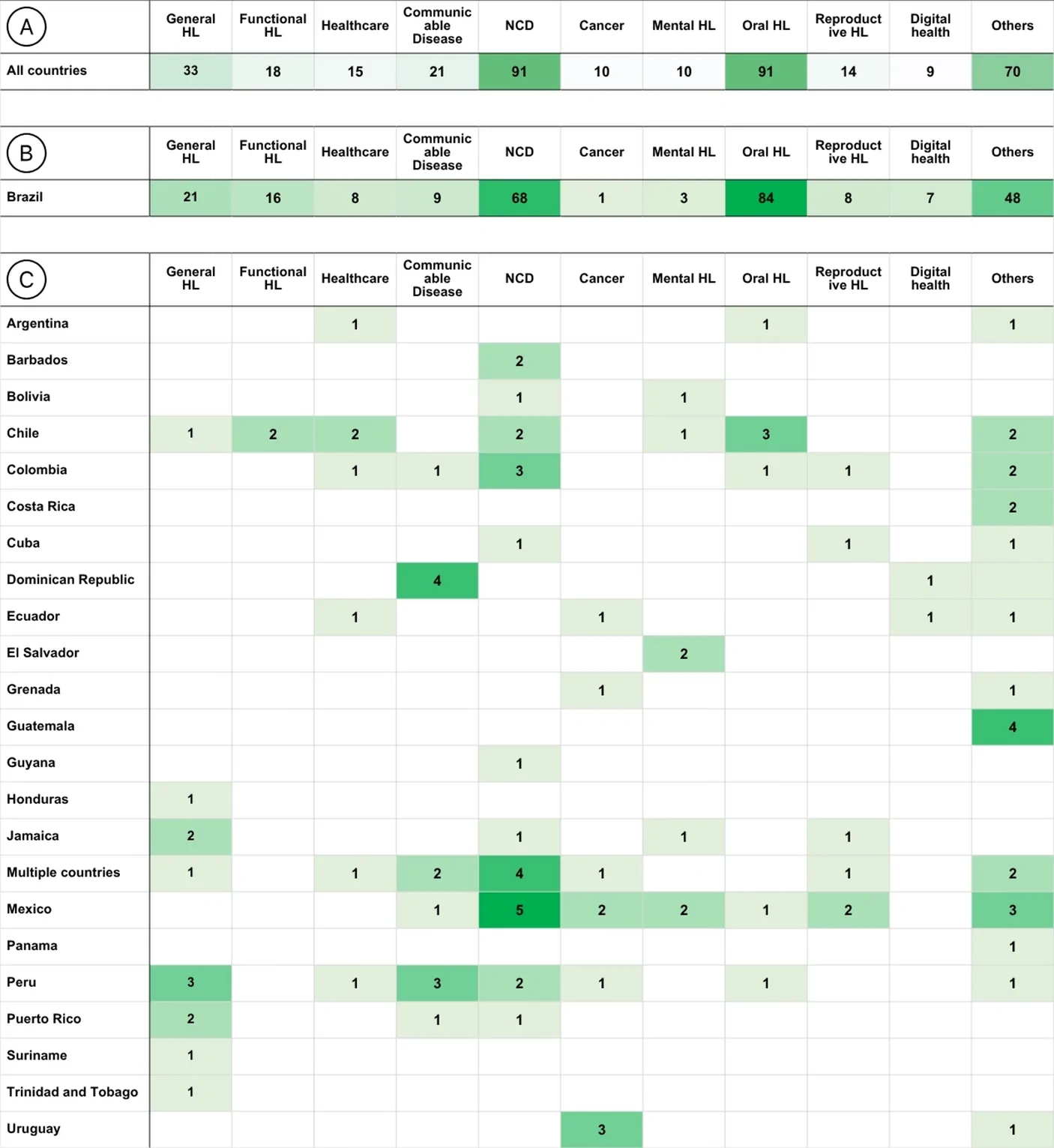
Heat map of health literacy topics grouped by major themes and subthemes. A: across Latin America and the Caribbean, B: in Brazil, D in Latin America and the Caribbean excluding Brazil

**Fig. 5:**
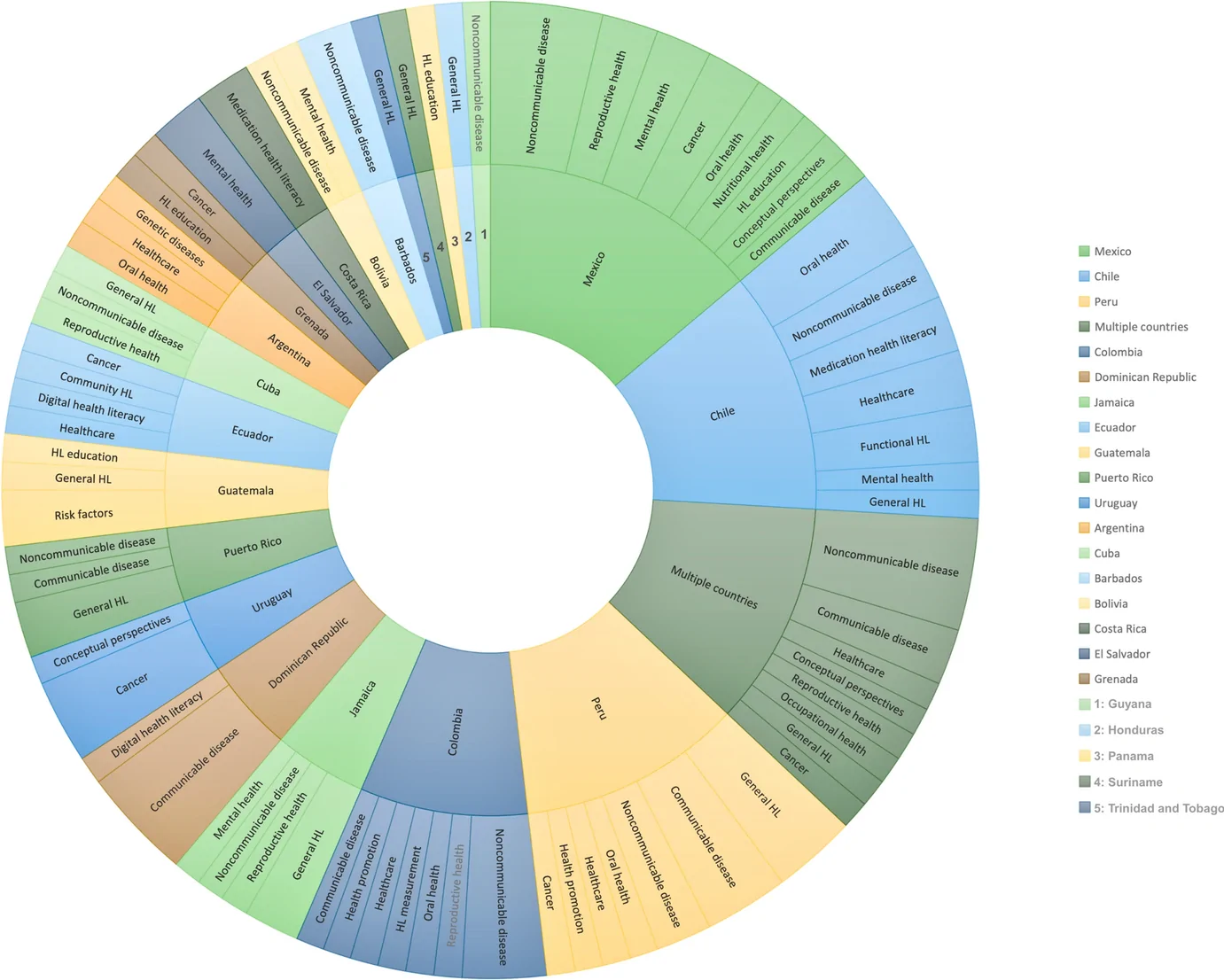
Overview of distribution of research topics per country in Latin America and the Caribbean excluding Brazil

The analysis revealed that the two most frequently studied topics were oral health literacy (n = 91, of which 84 were from Brazil) and non-communicable diseases (n = 85, of which 63 were from Brazil). Other frequently studied topics included general health literacy (n = 33), health literacy and communicable diseases (n = 21), functional health literacy (n = 17), and healthcare literacy (n = 15). Additional topics addressed included health literacy in relation to reproductive health, digital health, mental health, and cancer. The less frequently studied topics were grouped as ‘other’ in the heat maps in Fig. 4, such as medication or health promotion-related health literacy. The heat maps show the topical research patterns across all countries in Latin America and the Caribbean (Fig. 4A), in Brazil (Fig. 4B), and across Latin America and the Caribbean excluding Brazil (Fig. 4C). Figure 5 shows a comprehensive overview of the distribution of research topics in Latin America and the Caribbean with the exclusion of Brazil. Further details about the coding scheme can be found in Supplement 5.

### Review of Measurements

The sample reported that a total of 312 times instruments were used to measure health literacy. A significant number of studies have employed two or more instruments to evaluate health literacy, particularly those that validated translated instruments. The majority of these instruments were developed outside Latin America and the Caribbean, predominantly in European countries and English-speaking nations such as the USA and Australia.

**Fig. 6:**
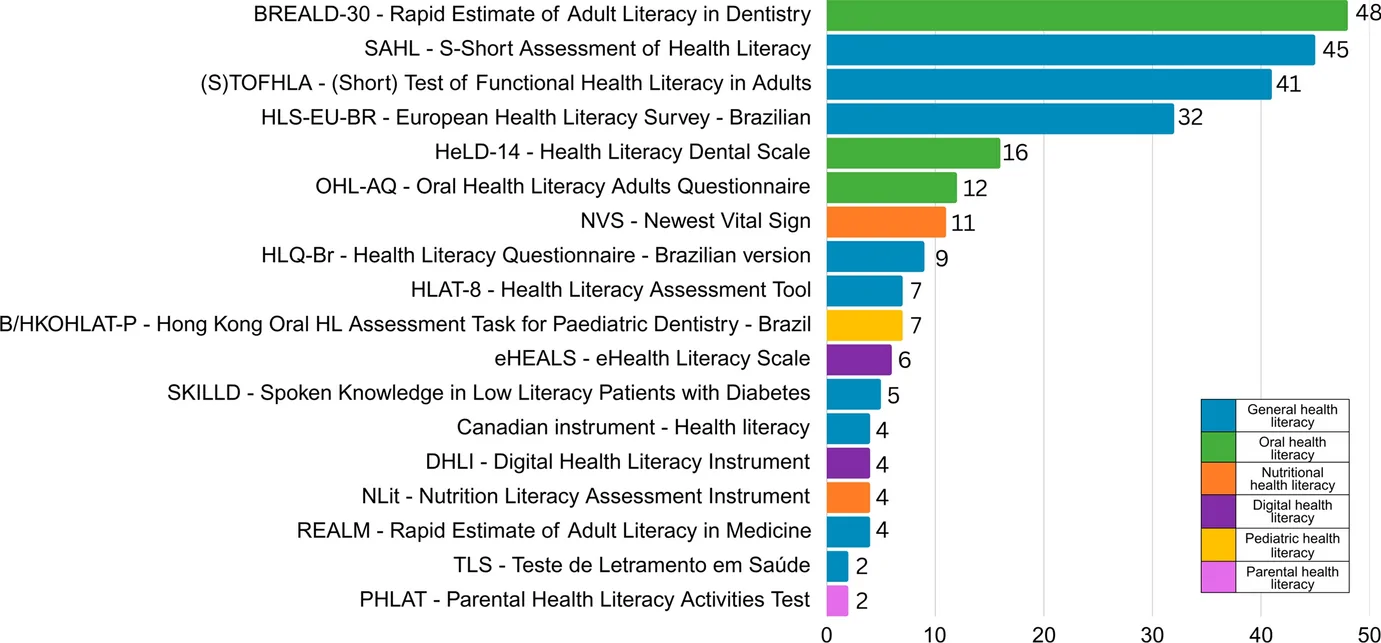
Health literacy measurements used in Latin American and Caribbean research

Eighteen distinct instruments or instrument families were utilized two or more times to assess health literacy, whereas 31 instruments were utilized only once. The four most frequently used tools were the BREALD-30 (Rapid Estimate of Adult Literacy in Dentistry), SAHL-S (Short Assessment of Health Literacy), S-TOFHLA (Short Test of Functional Health Literacy in Adults), and HLS-EU (European Health Literacy Survey). The most frequently measured health literacy topics entailed general health literacy (n = 149), oral health literacy (n = 76), nutritional health literacy (n = 15), digital health literacy (n = 10), and pediatric-related health literacy (n = 7). For a comprehensive list of the most used instruments, refer to Supplement 6. Other measurements covered specific topics such as diabetes, chronic kidney disease, smoking, elderly care, the Coronavirus (SARS-CoV-2), the Human Immunodeficiency Virus (HIV), and cancer. (Fig. 6).

## Discussion

This scoping review examined how health literacy research has evolved in Latin America and the Caribbean with regard to the prevailing historical trends, geographical distribution, use of definitions, research topics, and assessment methods. The findings provide a timely and comprehensive synthesis of the current landscape of health literacy research in the region, including valuable insight into the scope, methodological diversity, and topical orientations of scholarly activity.

### Study Design

The study revealed that there was a pronounced inclination towards numerical data, standardized instruments, and statistical analysis in comparison with other methods. Although most studies employed quantitative methods, the presence of qualitative and mixed-method designs suggests a gradual diversification of methodological approaches, which have the potential to offer a more holistic understanding by combining numerical trends with contextual explanations (Wasti et al., 2022). However, despite the proliferation of research on health literacy in Latin America and the Caribbean, there was a paucity of interventions and longitudinal studies according to this review. A tendency that was also documented by Paucar-Caceres et al. (2023). Moreover, the studies focusing on organizational health literacy—the capacity to recognize, prioritize, and sustain actions aimed at improving health literacy across systems — was seemingly scarce, perhaps because it is a rather new area of research interest around the globe (Agency for Healthcare Research and Quality et al., 2012; Trezona et al., 2017). The observed discrepancy between academic productivity and practical implementation of health literacy evidence at community, organizational, and system levels underscores the pressing need for structural and institutional changes that not only facilitate knowledge generation but also ensure its translation into real-world health contexts (Batterham et al., 2016). Nonetheless, the data sample deliberately included peer-reviewed publications as well as dissertations and theses, visibly showcasing a new generation of health literacy research in the pipeline, which is likely to support the further publication and dissemination of health literacy research in the future to inform and inspire policy and practice.

### Historical Trends

The number of publications originating from Latin America and the Caribbean has increased considerably in recent decades. The initial wave from 2009 to 2017 was likely influenced by developments in North America, such as the influential work of the Institute of Medicine and the Obama administration, which promoted health literacy, including the establishment of the National Action Plan on Health Literacy in the United States (U.S. Department of Health and Human Services, Office of Disease Prevention and Health Promotion, 2010). Moreover, it is clear from the list of authors engaged in the research scrutinized in the review that many American researchers, research institutions, and networks contributed to knowledge-transfer on health literacy in Latin America and the Caribbean. This is much in line with PAHO’s general call for knowledge-transfer to form evidence-based health policy, research and practice development in the region (Pantoja et al., 2018). The increase in publications from 2017 onward may be associated with the rise of the global health literacy movement (Sørensen et al., 2018) including the prominent role of health literacy in the 9th Global Conference on Health Promotion in Shanghai (WHO, 2016) positioning health literacy as a critical pillar for achieving the Sustainable Development Goals and the launch of the International Health Literacy Association in 2016. Moreover, the accelerated progression and proliferation of digital technologies have facilitated expedited access to information and innovative health communication methodologies, thereby catalyzing a global demand to enhance health literacy (van Kessel et al., 2025). In addition, the Coronavirus (SARS-CoV-2) pandemic increased the awareness of the need for health literacy to manage the outbreak at the individual and societal levels (Teixeira Netto et al., 2022). Regarding the drop in publications on health literacy in Latin America in 2023 several explanations may be likely. It can be due to bibliometric fluctuation rather than a true reversal of interest. However, it might also be due to the “post-pandemic normalization” which is reflected in broader regional science trends where research publications from some fields plateau or temporarily dip after COVID-driven peaks (Vinadé Chagas et al., 2025; Zabala et al., 2023).

### *Geographical distribution:*

 Health literacy research was identified in two-thirds of the countries in Latin America and the Caribbean. Brazil accounted for the highest number of publications in the region. However, one in three countries has yet to publish any health literacy research. These countries include Belize, Nicaragua, Antigua and Barbuda, the Bahamas, Dominica, Haiti, Kitts and Nevis, Saint Lucia, Vincent and the Grenadines, Paraguay, and Venezuela. A plethora of factors may impede a nation’s initiation of health literacy research, including, but not limited to: prevailing policies, research traditions, competing priorities, and constrained resources. As observed in this region and in other parts of the world, assessing health literacy represents a critical initial step in establishing the need to address it, encouraging the development of interventions, and formulating guidelines and policies to enhance individual and organizational health literacy (Sørensen et al., 2021).

Brazil’s leading position may be attributable not only to the country’s substantial geographical expanse but more crucially, to its strong academic engagement in health literacy research, as evidenced by the high number of publications, theses, and dissertations identified. The finding is consistent with the results of previous bibliometric analyses, which have also underscored Brazil’s significant contributions to this region’s health literacy research (Arrighi et al., 2022; de Jesus et al., 2024; Lima et al., 2025). The concomitant rise in studies conducted in Brazil following the establishment of the Brazilian Health Literacy Network (REBRALS) (Brazilian Health Literacy Network, 2026) is not coincidental. This initiative, launched in late 2019, was spearheaded by researchers committed to consolidating fragmented knowledge and efforts, with the aim of producing studies that would emphasize the urgency of investment in health literacy research (Peres et al., 2023). However, the absence of national surveys on health literacy and the limited integration of the concept into public policy illustrate the marginal position health literacy occupies within Brazil’s general health agenda (Moraes, 2018). Scholars such as Moraes have argued that "Brazil needs to be awakened to the health literacy issue and understand that its incorporation into health practices can create important opportunities for the development of a universal, comprehensive, and equitable health system." (Moraes, 2018).

### Health Literacy Descriptions and Definitions

More than 75% of the publications made use of internationally recognized references in primary or secondary literature. These definitions have emerged organically in different regions worldwide, often influenced by local priorities such as patient education, public health, or healthcare navigation (Sørensen et al., 2012). When the conceptual underpinnings are described, it makes it easier to understand the rationale and perspectives provided in the publications. It is particularly important with regard to translations of the term health literacy in other languages than English, as translations are influenced by disparate sociocultural contexts and varying academic discourses, each with its own unique development (Hernández-Rivera et al., 2022; Sørensen & Brand, 2013). In the Spanish context, for example, *alfabetización* is often associated with basic abilities (Sørensen & Brand, 2013). In the context of Brazilian Portuguese, a variety of terms have been identified as referring to health literacy. However, it is crucial to note that education researchers have explicitly delineated the distinction between "alfabetizar," which signifies the process of imparting fundamental reading and writing abilities, and "letrar," which conveys the development of wider literacy skills (Soares, 2002, 2022). These sorts of translational challenges are not unique to Latin America and the Caribbean; they are also found in Europe and beyond (Sørensen & Brand, 2013).

### Topical Review

There were marked topical differences in health literacy research across Latin America and the Caribbean. A majority of studies focused on oral health literacy and non-communicable diseases, with Brazil contributing the most publications in these fields in comparison to other topics, such as general health literacy, functional health literacy, and healthcare literacy as well as digital health, mental health, and reproductive health. These findings differ from other regions, e.g., Africa, where research on oral health literacy is less prominent and e.g. instead mental health literacy and reproductive health literacy play a stronger role than in Latin America and the Caribbean (Sørensen et al., 2024). This variability underscores the context- and content-specific nature of health literacy, highlighting the importance of adopting nuanced approaches to its measurement, assessment, intervention, and policy formulation (Sørensen et al., 2021).

### Review of Measurement

The review identified a wide range of health literacy measurement tools, including both established instruments and newly developed ones. More than 15 instruments were created by either developing new items or adapting pre-existing tools, often without reporting on an adequate assessment of their psychometric properties. While it is a sign of innovation and attempts to adapt the tools to local needs, it may also raise concerns regarding methodological rigor and consistency (Tavousi et al., 2022). Without proper evaluation of validity and reliability, the accuracy of the results generated by these instruments is uncertain (American Educational Research Association, 2011; Boateng et al., 2018). More research is warranted to strengthen the assessment of health literacy in the region, taking into account the importance of developing tools grounded in the perceptions and lived experiences of the target populations to enhance validity and relevance (Batterham et al., 2016; Haun et al., 2014). Without such cultural grounding, tools may fail to capture the specific challenges and realities faced by different communities. For example, significant variations exist between European Portuguese and Brazilian Portuguese, as well as between different variants of Spanish, such as those spoken in Mexico and Argentina. Hence, the development and application of health literacy instruments require careful attention to socio-linguistic, systemic, and cultural differences (Boateng et al., 2018).

### Diverse Population Needs

Although not presented in detail, identified pivotal barriers, including socioeconomic inequality and cultural and linguistic differences, persist in affecting people in vulnerable circumstances, such as indigenous and rural populations, in a disproportionate manner (Bancalari et al., 2022; Campero et al., 2014; Mora Vicarioli et al., 2021), according to this study. These challenges in health literacy research are further compounded by the high levels of migration across Latin America (Avis, 2024; Blyde et al., 2020; World Health Organization, 2022). Population mobility introduces additional layers of cultural and linguistic diversity that complicate both healthcare delivery and the design of standardized measurement tools and interventions. The societies in Latin America and the Caribbean in the twenty-first century are increasingly diverse, often encompassing individuals with distinct cultural backgrounds, native languages, and health-related beliefs and experiences. As a result, measuring complex constructs such as health literacy requires tools that are both sensitive to context and adaptable across populations.

### Health Education in Healthcare and Communities

A significant proportion of the research included in the scoping review was conducted in healthcare settings as well as in communities with a pronounced emphasis on health education and popular education, which is a distinct branch of education within the Latin American context. The prevailing approach in healthcare and community health is rooted in Freire’s philosophy of health education and popular education (Freire, 2005), exhibiting notable parallels with the conceptualization of health literacy in numerous aspects. Consequently, the adoption of health literacy should not be perceived as a threat but rather as an opportunity to enhance and expedite ongoing initiatives (Abel & Benkert, 2022). Furthermore, examining the theories and pedagogical approaches underlying the various health literacy and health education interventions (see Sarr et al., 2025) could provide a valuable foundation for further policy, practice, and research development.

### Strengths and Limitations

A key strength of this scoping review lies in the meticulous and systematic formulation of the search strategy and the thorough identification of relevant records. The review encompassed a wide range of databases at both global and regional levels, capturing a broad spectrum of academic output, including peer-reviewed journal articles, theses, and dissertations. This inclusive approach enabled a comprehensive overview of the existing scientific literature on health literacy within Latin America and the Caribbean.

Another notable strength is found in the analytical process undertaken by the research team. Comprising four researchers from diverse academic backgrounds, the team applied a structured analytical framework to explore developments in health literacy from multiple angles. The interdisciplinary nature of the team not only provided complementary expertise but also strengthened the interpretative depth of the findings.

Nevertheless, several limitations must be acknowledged. This study encompasses the extensive corpus of research literature published during the period spanning from the initial mention of health literacy in 2009 to January 2024. Backward citation search and search for grey literature was not conducted. The classification of certain publications posed challenges, as not all could be easily assigned to the predefined categories. In such cases, final decisions were made through group discussion to reach consensus. A further constraint stems from linguistic variation. While the review included studies published in English, Spanish, and Portuguese, relevant articles published in French, particularly those possibly addressing Caribbean or Latin American issues, were not identified. Importantly, variations in the translation and use of the term “health literacy” across languages may have affected the retrieval of relevant material. Studies addressing related constructs like *information literacy*, *health knowledge*, *bi-alfabetización en salud reproductiva*, or *alfabetización sanitaria* were deliberately excluded if they did not employ the term health literacy in any of the included languages, despite their relevance to maintain a strict focus on the research objective, which concerned mapping relevant health literacy research developments. Finally, limited access to certain publications, theses, and dissertations may have affected the comprehensiveness of the review. Many of these documents are housed exclusively in institutional repositories and are not openly accessible, which likely led to an underestimation of the actual volume of health literacy research. More research is warranted to follow progress and expand the study in the future. Nevertheless, this review constitutes a unique and valuable baseline and foundation upon which future research may be built.

## Implications for the Advancement of Health Literacy in Latin America

Complementing previous regional reviews (Arrighi et al., 2022; Jesus et al., 2024; Lima et al., 2025; Paucar-Caceres et al., 2023), this comprehensive scoping review underscores both the progress and persistent challenges in advancing health literacy across Latin America and the Caribbean and contributes to the existing body of evidence on health literacy from regional perspectives in Africa, Asia, and Europe (Duong et al., 2017; Sørensen et al., 2015, 2024). While regional health literacy assessment has been a strong driver of the health literacy agenda in Asia and Europe, there is no regional survey involving the countries in Latin America and the Caribbean. Conversely, the impetus for research endeavors is predominantly derived from national and local stakeholders. In contexts such as Latin America and the Caribbean, where the health literacy agenda is emerging, institutional support is crucial to ensure scientific rigor and long-term impact. In that regard, community engagement and multidisciplinary approaches are essential for tailoring health literacy initiatives to diverse populations. Policy recommendations emphasize the need for coordinated, multisectoral action involving governments, healthcare professionals, educators, the private sector, and community organizations. The evaluation and monitoring of health literacy levels represents merely the initial phase of a multifaceted process; it is imperative that subsequent endeavors focus on the formulation of policy- and practice-oriented strategies, which are required to translate the findings into tangible actions such as curriculum development, policy action plan (M-POHL, 2023). A more profound examination of the function of health literacy in science communication, with a particular emphasis on the manner in which it enables the dialogue between research, practice and policy, could yield additional pertinent insights (see Ishikawa & Kiuchi, 2010; Ratzan, 2001).

A key strategy could be to increase organizational and system health literacy (Agency for Healthcare Research and Quality et al., 2012; Sørensen et al., 2021), which is found to bridge inequality and contribute to better health service delivery. Another would be to design national health literacy strategies and action plans to advance the quality of care and community health and address the challenges compounded by the limited resources available in healthcare systems, the complexity of these systems, and the lack of assessments and interventions tailored to local languages and contexts. This comprehensive scoping review identified promising evidence that can inform strategies and interventions to overcome barriers and empower patients and other population groups.

Looking ahead, there is a clear need to continue the progress on health literacy research in Latin America and the Caribbean, as well as for the integration of health literacy principles into education, healthcare, and public policy. Strengthening collaboration between international organizations, ministries of health and education, academic institutions, and community organizations, expanding access to culturally appropriate resources and fostering community champions are critical steps toward reducing disparities and promoting health equity through the advancement of health literacy.

In conclusion, enhancing health literacy is a vital, modifiable social determinant of health that can promote individual autonomy and empowerment, improve health service delivery, reduce health inequities, and contribute to the sustainability of public health systems in Latin America and the Caribbean. It is imperative that ongoing research, policy innovation, academic engagement, and community-based action be undertaken to achieve the full potential of health literacy as a catalyst for enhanced quality of care, health and well-being throughout the region.

## Data Availability

The included articles were all available online and the references can be found in the supplementary material.
